# Associations between History of Hospitalization for Violence Victimization and Substance-Use Patterns among Adolescents: A 2017 Korean National Representative Survey

**DOI:** 10.3390/ijerph15071543

**Published:** 2018-07-20

**Authors:** Yeji Lee, Kang-Sook Lee

**Affiliations:** 1 Department of Public Health, Graduate School, The Catholic University of Korea, 222 Banpo-daero, Seocho-gu, Seoul 06591, Korea; fina@catholic.ac.kr; 2 Department of Preventive Medicine, College of Medicine, The Catholic University of Korea, 222 Banpo-daero, Seocho-gu, Seoul 06591, Korea

**Keywords:** adolescent, substance use, tobacco use, alcohol use, drug use, violence victimization

## Abstract

Violence victimization can adversely affect adolescents’ long-term health. Existing research has mainly focused on the link between victimization and substance use; however, the evidence obtained to date has been inconsistent. This study, using a Korean national representative sample, examined the association between violence victimization and substance-use patterns (including tobacco, alcohol, and drug use) in terms of sex and number of violence victimization experiences. We analyzed secondary data from the 2017 Korean Youth Risk Behavior Web-based Survey. Chi-squared test analyses and logistic regression analysis were used to examine substance use in terms of violence victimization; additionally, *p*-values for trends were calculated to reveal the dose-response relationship per number of violence victimization experiences. We consequently found that participants’ rates of tobacco, alcohol, and drug use were higher among those who experienced violence victimization than among those who did not. For each substance-use-related variable, the greater the violence victimization experience, the higher the odds of substance use (*p* for trend < 0.001). Early intervention may help prevent the development of substance use, especially among adolescents who have experienced violence victimization. Prevention efforts regarding substance abuse and violence prevention should be included in school curricula to effectively prevent adverse health consequences among adolescents.

## 1. Introduction

Violence victimization is a broad, bridging topic that includes physical harm, emotional and psychological distress, systemic social injury, and gender discrimination [1]. Over half of all children in the world have been exposed to violence, with the highest number of youths exposed to violence being located in Asian regions [2]. In Korea, the rate of school violence victimization is 18% among middle school students and 16.8% among high school students [3]. School bullying is one of the most common forms of violence faced by young people and is prevalent in almost every socioeconomic group in Korea [4]. According to the Rights of Children and Youths, physical violence, emotional violence, sexual violence, and neglect affect 37.0%, 21.9%, 3.4%, and 34.8% of Korean adolescents, respectively [5]. Violence victimization can adversely affect adolescents’ health, which may have long-term consequences [6]. In fact, adolescent exposure to violence victimization is associated with numerous health problems, such as physical injuries, asthma, rhinitis, unhealthy weight control behavior, obesity, depression, and suicidality [6,7,8,9].

Previous research has also focused on the link between involvement in victimization and substance use. In particular, this relationship is of great concern regarding adolescents; adolescence is a period during which individuals are especially vulnerable to substance use, as engaging in such practices at this developmental stage can increase the risk of developing many health problems and other issues, including personality disorders and poor academic achievement; further, such substance abuse or addiction can persist into adulthood [10,11,12]. According to the 2017 Korea Youth Risk Behavior Survey, 40.2% of adolescents in Korea have previously consumed alcohol, while similar use of tobacco, electronic cigarettes (ECs), and drugs is 13.7%, 7.4%, and 0.6%, respectively [13]. In addition, the prevalence of non-prescribed diet pill use ranges from 1.8 to 5.8%, which is another matter of great public health concern in Korea, due to adolescents’ unhealthy weight control behaviors [14]. These statistics suggest that the health risks that can arise from violence victimization and substance use can be a burden to public health.

Despite the increased attention being attributed to violence victimization, the relationship between violence victimization and substance use requires more in-depth analysis. It is somewhat difficult to compare related existing findings directly because this relationship is complex. Victimization can exist in many types (e.g., physical, mental, verbal, exclusion, etc.) [15,16,17]; for example, the environments and situations can differ (e.g., school, domestic, intimate violence, etc.), and individuals could be involved as pure victims or as both bullies and victims [17,18,19]. Further, different types of substances affect individuals differently, depending on their physical, psychological, gender, and cultural factors [9,20,21]. Current studies also show complex consequences of the relationship between victimization and substance use. Peer victimization was associated with smoking and alcohol abuse among Chinese adolescents [9]. Further, in the US, adolescents who were moderately bullied were found to show an increased risk of alcohol and cocaine use, but adolescents who were severely victimized were only associated with alcohol use [22]. Meanwhile, another study of US adolescents reported minor effects, such as cigarette smoking and alcohol use, on victims [18]. However, an examination of Australian adolescents found that being passive victims is not associated with substance use behavior [19]; further, Quinn et al. found no associations between victims and substance use among Australian adolescents [23].

Although Korean adolescents appear to have an elevated risk of being bullied [3,4,5], few studies have addressed the risk factors for violence victimization among Korean adolescents [4,8,24], and none have examined the association with substance use patterns. Therefore, in this study we examined this association, taking a Korean national representative sample and focusing on the relationship between tobacco, alcohol, and drug use and history of hospitalization for violence victimization, stratifying the results in terms of youths’ sex and the number of hospitalizations for violence victimization.

## 2. Materials and Methods

### 2.1. Study Population

This study analyzed secondary data from the 2017 Korean Youth Risk Behavior Web-based Survey (KYRBWS), which has been conducted annually by the Korea Centers for Disease Control and Prevention since 2005 to examine health behaviors, including smoking, alcohol intake, overweightness, dietary life, and physical activity, among Korean adolescents [13]. Students’ participation is voluntary, and participants’ responses to the self-administered online questionnaire questions were aggregated to preserve anonymity.

The 2017 KYRBWS data were collected in April 2017, and consisted of 123 questions across 15 health-related categories including smoking, alcohol consumption, physical activity, and mental health, etc.), with 107 indicators being calculated. The questionnaire items and indicators of KYRBWS are revised each year by a committee composed of experts from each relevant professional field, utilizing domestic and international data. Each question is considered reliable and valid [25]. To acquire representative Korean adolescent (13–18-years-old) samples, a complex sampling approach is used. This approach involves a three-stage random cluster sampling method. In the stratification stage, the study population is stratified by local group and school class, which serves to minimize sampling error. In the sample distribution stage, 400 middle and high schools were selected using the proportional distribution method, so that the population composition ratio by stratification variables and sample composition ratio were matched. For the stratified cluster sampling, the first extraction unit was the school, and the second extraction unit was the class. The sample schools are selected through the use of systematic sampling for each layer. Secondary extraction randomly extracts one class from the selected sample schools for each grade. Finally, in 2017, 799 schools and 62,276 students participated (response rate = 95.8%).

All 62,276 students who responded to the survey were included in this study, because there were no missing values except for the following variables: early initiation of smoking (8097 students provided adequate data; 54,126 students who had never smoked and 53 students who did not record when they first smoked were excluded), early initiation of EC use (4260 students provided adequate data; 57,974 students who had never used an EC, and 42 students who did not record when they first used an EC were excluded), and early initiation of drinking (24,355 students provided adequate data; 37,859 students who had never consumed alcohol and 62 students who did not record when they first consumed alcohol were excluded).

This survey was a government-approved statistical survey, and informed consent was obtained from all students who participated. This consent procedure was approved by the institutional review board of the Centers for Disease Control and Prevention of Korea (KCDC). We requested permission from the KCDC to use the KYRBWS data for the purpose of the research and made a pledge regarding its usage on the KYRBWS homepage. This study was approved by the institutional review board of the Catholic University of Korea (approval No. MC18QESI0029, date of approval—3 April 2018) to utilize secondary data of the 2017 KYRBWS.

### 2.2. Study Variables

#### Socio-Demographic Variables and Psychological Factors

Socio-demographic variables included sex, grade, residence area, perceived academic achievement, and perceived economic status. School grade was divided into first, second, and third grades in middle school, and first, second, and third grades in high school. Residence area was categorized as metropolitan city, city, and province. Perceived academic achievement was determined using the respondents’ own reports about their academic achievements in the past year, which was ranked using a five-point scale (high, middle high, middle, middle low, and low). Perceived economic status was determined using the respondents’ reports regarding their perceived house income level, again ranked using a five-point scale (high, middle high, middle, middle low, and low). The following psychological factors were taken into consideration as potential confounders: perceived stress, depression, and suicidal ideation [24,26]. Perceived stress was divided into five groups using the following question: “What is the usual level of stress you feel?”. Responses comprised very high, high, moderate, low, and very low. Depression was assessed using the question, “During the past year, have you ever felt too sad or desperate to perform normal daily activities for two consecutive weeks?”. Finally, suicidal ideation was assessed using the question, “Have you ever seriously thought about committing suicide in the past year?”.

### 2.3. Measurements

#### 2.3.1. History of Hospitalization for Violence Victimization

History of hospitalization for violence victimization was assessed using the following question: “How many times within the past 12 months have you received hospital treatment for violence victimization; for example, after experiencing physical violence; being threatened; or being ostracized by friends, seniors, or adults?”. The following responses were available to respondents: (1) never, (2) once, (3) twice, (4) three times, (5) four times, (6) five times, and (7) more than six times. Based on the responses, participants were categorized as non-victims (1) or victims (2–7). The number of participants who underwent hospitalization for violence victimization experiences was then categorized into four groups: none (1), 1–2/year (2–3), 3–4/year (4–5), and ≥5/year (6–7).

#### 2.3.2. Tobacco Use

Participants were determined to be previous smokers if they reported having smoked cigarettes before. Of these, those who had smoked within the past 30 days were categorized as current smokers; individuals who reported having smoked every day for the past 30 days were categorized as daily smokers; and those who had smoked over 20 cigarettes a day, on average, for the past 30 days were categorized as heavy smokers. Early initiation of smoking was evaluated among the previous smokers, and was defined as cases where participants had smoked before entering middle school. 

For EC, previous EC use was attributed to participants who had ever used ECs. Current EC use was then defined as having used ECs within the past 30 days, and daily EC use was defined as having used an EC every day for the past 30 days. Early initiation of EC use was evaluated among previous EC users, and was defined as cases where participants had used ECs before entering middle school.

#### 2.3.3. Alcohol Use

Participants who had consumed alcohol in situations other than performing ancestral rites, attending memorial ceremonies, or receiving Holy Communion were classed as previous drinkers. Current drinking was defined as consuming alcohol within the past 30 days, daily drinking was defined as having consumed alcohol every day for the past 30 days, and problematic drinking was indicated if the participants had consumed alcohol within the past 30 days and had answered “yes” to a series of at-risk assessment questions. To determine early initiation of drinking, the same protocol as that used for tobacco use was applied.

#### 2.3.4. Drug Use

Previous drug use was attributed to participants who had ever used drugs, inhaled butane gas, or sniffed glue habitually or intentionally. Current drug use was defined as currently using drugs such as butane gas, glue, stimulants, methamphetamine, amphetamines, overdosing on cold medicine or tranquilizers, with the purpose of achieving mood variation, hallucinatory experiences, or extreme dieting. Meanwhile, non-prescribed diet-pill use was attributed to participants who had attempted to control their weight by taking a diet pill arbitrarily, without a doctor’s prescription, within the past 30 days.

### 2.4. Statistical Analyses

All statistical analyses were performed using SPSS 25.0 (IBM, Armonk, NY, USA), which helped to reflect complex sampling weights.

An χ^2^ test analysis was used to compare participants’ general characteristics and psychological factors in terms of violence victimization, and to measure the prevalence of substance use among violence victims in terms of sex and the number of violence victimization experiences. A logistic regression analysis was conducted to calculate odds ratios and 95% confidence intervals, using the independent substance use variables as main outcome variables. These analyses were conducted after adjusting for psychological factors such as perceived stress, depression, and suicidal ideation, which can cause significant differences in violence victimization and socio-demographic variables. *p*-values for trends were performed to identify any dose-response relationship with the number of violence victimization experiences. Statistical significance was set at *p* < 0.05.

## 3. Results

### 3.1. Participants’ General Characteristics

Table 1 shows participants’ socio-economic variables and psychological factors, stratified in terms of history of hospitalization for violence victimization. The proportion of victims was 70.5% in boys and 29.5% in girls (*p* < 0.001). The proportion of participants who reported very high perceived stress was higher among victims than among non-victims (*p* < 0.001). In addition, the proportion of participants who reported awareness of depression was higher among victims than among non-victims (*p* < 0.001). Regarding suicidal ideation, the proportion of participants with suicidal ideation was 11.4% among non-victims and 35.3% among victims (*p* < 0.001).

### 3.2. Prevalence of Tobacco, Alcohol, and Drug Use among Korean Adolescents Who Have Been Hospitalized for Violence Victimization, Stratified in Terms of Sex

Table 2 shows substance use among participants with a history of hospitalization for violence victimization, stratified in terms of sex. For boys, the prevalence of current smoking, daily smoking, and heavy smoking was 27.4%, 11.0%, and 9.3%, respectively, while for girls, the prevalence was 24.9%, 8.1%, and 8.1%, respectively. Female victims showed a higher prevalence of current EC use and daily EC use than male victims (current EC use: 19.4% vs. 19.3% and daily EC use: 6.0% vs. 5.6%). Female victims also showed a higher prevalence of previous drinking, daily drinking, and problematic drinking than male victims (previous drinking: 55.4% vs. 53.8%, daily drinking: 6.5% vs. 6.4%, and problematic drinking: 21.3% vs. 19.0%). In addition, female victims showed a higher prevalence of previous drug use and current drug use than male victims (previous drug use: 17.9% vs. 15.4% and current drug use: 7.5% vs. 5.4%). Prevalence of non-prescribed diet pill use among victims was higher in boys (11.1%) than in girls (9.3%).

### 3.3. Prevalence of Tobacco, Alcohol, and Drug Use in Terms of Number of Hospitalizations for Violence Victimization

Table 3 shows substance use in terms of number of hospitalizations for violence victimization. It can be seen that for all tobacco-use variables, the prevalence increases as the number of hospitalizations for violence victimization increases (*p* < 0.001). This is also the case for the alcohol-use variables (*p* < 0.001) and drug-use variables (*p* < 0.001).

### 3.4. Association between Substance Use and History of Hospitalization for Violence Victimization in Terms of Sex

The association between substance use and history of hospitalization for violence victimization among adolescents is shown in Table 4, stratified by sex. The odds ratios (ORs) for current smoking, heavy smoking, and current EC use were 3.44, 10.52, and 6.70, respectively, for male victims, while the ORs were 6.18, 11.94, and 15.39, respectively, for girls. The ORs for current drinking, daily drinking, and early initiation of drinking were 2.07, 8.71, and 3.59, respectively, for male victims, while the ORs were 2.51, 17.53, and 5.76, respectively, for girls. In addition, the ORs for previous drug use, current drug use, and non-prescribed diet-pill use were 11.55, 11.88, and 14.26, respectively, for male victims, while the ORs were 13.78, 14.45, and 3.57, respectively, for girls.

### 3.5. Association between Substance Use and Number of Hospitalizations for Violence Victimization

The associations between substance use and number of hospitalizations for violence victimization among Korean adolescents are shown in Table 5. The ORs for heavy smoking were 32.51, 14.60, and 4.51 for participants who reported being bullied more than five times per year, three to four times per year, and one to two times per year, respectively (*p* for trend < 0.001). The ORs for daily EC use were 21.16, 6.23, and 2.70 for participants who had been bullied more than five times per year, three to four times per year, and one to two times per year, respectively (*p* for trend < 0.001). The ORs for daily drinking were 36.44, 7.54, and 3.65 for participants who was been bullied more than five times per year, three to four times per year, and one to two times per year, respectively (*p* for trend < 0.001). In addition, the ORs for current drug use were 29.68, 18.24, and 6.03 for participants who had been bullied more than five times per year, three to four times per year, and one to two times per year, respectively (*p* for trend < 0.001).

## 4. Discussion

This study investigated the relationship between violence victimization and substance use patterns among Korean adolescents using national representative data. Our main finding is that there is a significant association between history of hospitalization for violence victimization and substance use patterns. The higher the number of hospitalizations for violence victimization, the higher the odds ratio for each substance-use-related variable (*p* for trend < 0.001).

One possible explanation for victims’ substance use is that victims use substances as a defense against threatening situations. For example, many victims may use cigarettes or alcohol as coping methods for bullying in order to minimize fear, distress, or shame [9,27,28]. Involvement in victimization can cause adolescence to have interpersonal difficulties such as elevated distress, anxiety, depression, and emotional difficulties [29,30,31]. Interpersonal difficulties among adolescents lead to substance use, because adolescents have not developed appropriate coping skills yet [32]. Thus, it can be assumed that substance use may be a result of the continuing stress or depressive mood by victimization, which can be explained in the elevated levels of substance use among victims [28]. Interventions aimed at developing appropriate management strategies for these interpersonal difficulties instead of negative or immature copying styles would be best targeted at adolescent victims. 

Alternative explanations, such as the possibility that adolescents may use smoking and drinking behaviors to improve the social image they present to their peers [33], must also be considered. Adolescent smokers have a desire to be considered attractive, powerful, and “cool” [34]; therefore, victims believe that they will be bullied less if they engage in a rebellious act such as smoking. Further, victims of bullying often lack friends and many tend to be rejected by their peers [35,36], inferring that some adolescent victims use substances as a means of gathering and socializing with others in order to join peer groups, thus avoiding victimization. Future research should work toward establishing firm evidence to describe the related factors affecting substance use in victims such as coping skills or social relationship.

Our study showed that associations between victimization and substance use such as current smoking, current EC use, current drinking, problematic drinking, and current drug use were stronger among females than among males. Concurrent with our study, Guo’s study of Chinese adolescent samples revealed that female victims are more likely to be current smokers [28]. These results are consistent with those of a previous study that examined a US adolescent population [37]. Thus, it can be inferred that many girls do not have effective strategies for coping with bullying, since bullying can be an extreme or rare situation for girls [37], in turn making it more likely to contribute to substance use. Specifically, it could be differentiated by forms of aggression: Tharp-Taylor et al. demonstrated that adolescent male victims risk developing an association with alcohol, marijuana, and inhalant use when they experience mental bullying, whereas adolescent female victims become sensitive to substance use when they are exposed to physical bullying [15]. This is because males are relatively familiar with physical violence, but are unfamiliar with mental violence, whereas females are relatively more commonly engaged in more mental violence, but are not accustomed to physical violence. Adolescents tend to use more substances under less familiar circumstances [15]. Clearly, greater understanding of why sex influences substance use differently is needed.

To our knowledge, this is the first assessment of the relationship between violence victimization and EC and non-prescribed diet-pill use. Nicotine administration using ECs may result in abnormal serotonergic activity, which can be associated with impulsive or aggressive behaviors and violence-related behaviors [24,38,39]. Considering that developing adolescents are more vulnerable to nicotine addiction, which affects cognitive function [40], and some prior studies [41,42,43] have identified a relationship between EC use and mental-health conditions, the possibility that the use of ECs may function as a gateway to future drug use is a real danger for adolescent victims [38], particularly adolescent EC users who have mental health problems due to violence victimization. 

The problematic behavior of non-prescribed diet-pill use may infer that adolescent victims have a false sense of weight control. Overweight or obese children are more likely to be bullied than normal-weight children as a result of weight stigmatization [44], eventually resulting in adverse outcomes such as psychological problems, low self-esteem, body dissatisfaction, and avoidance of health care [45]. A study of South Korean primary students found that victimization behaviors are associated with poorer body satisfaction [46]. Meanwhile, Jackson showed that there is a link among youths between unhealthy diet and victimization [47]. In conclusion, improving dietary habits may be a crucial point of intervention in efforts to minimize the improper use of diet pills, which could consequently improve adolescent health through indirectly reducing the prevalence of the negative health consequences of bully victimization.

### Limitations and Strengths

The present study has some limitations to note. First, the cross-sectional nature of this study limited the ability to draw causal conclusions. Future longitudinal studies are needed to determine that victimization leads to substance use, rather than vice versa. Second, our study may have included bullies/victims in the victim group, since we could not control for other bullying role behaviors. Given that adolescents can be involved in multiple bullying behaviors and that victimization was found not to be significantly related to substance use after controlling for bullying role behaviors [23], emphasizing the importance of controlling for other bullying role behaviors is essential. Third, our study used one incorporated question to measure history of hospitalization for violence victimization, which failed to assess substance use in terms of distinct types of victimization behaviors, such as verbal, physical, relational, and cyber. Hospitalization for violence victimization can be somewhat ambiguous, as it is difficult to precisely define violence; our measurement of hospitalization may have included psychological treatment as a result of being ostracized by others, which can be related to mental trauma. Future research is required to determine if the findings are consistent across the diverse types of victimization behaviors. Fourth, this study relied on a self-report design; therefore, self-presentation bias may exist. Adolescents can underreport their substance use because of social desirability, and the number of hospital treatments for violence victimization in the past year could also be incorrectly reported. Fifth, our study was unable to include out-of-school youths because the survey was conducted on middle school and high school students. Considering that out-of-school youths have an elevated risk of developing substance disorders [48] and involvement in violence [49], future research that includes out-of-school youths is warranted, and this would increase the validity of this study.

Despite these limitations, our study possesses the following noteworthy strengths. To the best of our knowledge, this was the first study to use a nationally representative sample to examine the association between violence victimization and substance use in Korean adolescents. In addition, our study assessed not only the prevalence of substance use, but also other substance-use-related variables, including EC use and non-prescribed diet-pill use, which have never previously been researched. Furthermore, in order to obtain reliable results, this study analyzed and adjusted variables, including psychological variables, which might have affected or be related to substance use in adolescents.

## 5. Conclusions

By showing the presence of a significant association between violence victimization and risk of substance use, our study suggests that victims of violence should be considered priority targets for prevention programs for substance use. In accordance with previous research that demonstrated the importance of using appropriately designed programs to prevent substance use in adolescents [50,51,52], professionals engaging in substance use prevention should consider victims’ characteristics in depth. Our findings highlight the importance of, when designing and offering intervention programs aimed at adolescent substance users, considering the increased risk associated with adolescents who are bullied. Further, efforts to address substance abuse and violence prevention should be included as part of school curricula in order to effectively prevent adverse health consequences in adolescents. Above all, considerable effort is needed to reduce violence victimization among adolescents, because reducing violence is one of the most important methods of reducing substance use and other risk factors. 

## Figures and Tables

**Table 1:** Participants’ general characteristics (*n* = 62,276).

Variables	Category	All Participants	Non-Victim	Victim	*p*
Sex	Boys	31,624 (52.1)	30,466 (51.6)	1158 (70.5)	<0.001
	Girls	30,652 (47.9)	30,134 (48.4)	518 (29.5)	
School grade	Middle school 1st grade	10,189 (14.8)	9959 (14.9)	230 (13.0)	<0.001
	Middle school 2nd grade	10,377 (15.4)	10,044 (15.3)	333 (18.1)	
	Middle school 3rd grade	10,319 (15.1)	9968 (14.9)	351 (19.8)	
	High school 1st grade	10,165 (17.1)	9958 (17.2)	207 (12.9)	
	High school 2nd grade	10,800 (19.0)	10,528 (19.0)	272 (17.6)	
	High school 3rd grade	10,426 (18.6)	10,143 (18.6)	283 (18.7)	
Residential area	Metropolitan city	27,629 (43.1)	26,873 (43.0)	756 (44.5)	0.637
	City	29,808 (50.8)	29,020 (50.8)	788 (49.8)	
	Province	4839 (6.1)	4707 (6.1)	132 (5.7)	
Perceived academic achievement	High	8528 (13.6)	8172 (13.4)	356 (22.0)	<0.001
	Middle high	15,996 (25.6)	15,655 (25.7)	341 (21.3)	
	Middle	17,810 (28.7)	17,445 (28.9)	365 (21.8)	
	Middle low	13,818 (22.2)	13,497 (22.3)	321 (17.8)	
	Low	6124 (9.9)	5831 (9.7)	293 (17.1)	
Perceived economic status	High	6713 (11.0)	6341 (10.6)	372 (23.0)	<0.001
	Middle high	18,089 (29.4)	17,694 (29.6)	395 (24.0)	
	Middle	28,582 (45.6)	28,043 (46.0)	539 (31.6)	
	Middle low	7299 (11.5)	7089 (11.5)	210 (11.8)	
	Low	1593 (2.6)	1433 (2.4)	160 (9.6)	
Perceived stress	Very high	6508 (10.3)	6110 (9.9)	398 (23.2)	<0.001
	High	16,751 (26.9)	16,335 (26.9)	416 (24.7)	
	Moderate	26,271 (42.6)	25,738 (42.9)	533 (31.5)	
	Low	10,229 (16.2)	10,040 (16.4)	189 (11.8)	
	Very low	2517 (4.0)	2377 (3.8)	140 (8.7)	
Depression	No	46,664 (74.9)	45,840 (75.6)	824 (48.8)	<0.001
	Yes	15,612 (25.1)	14,760 (24.4)	852 (51.2)	
Suicidal ideation	No	54,692 (87.9)	53,613 (88.6)	1079 (64.7)	<0.001
	Yes	7584 (12.1)	6987 (11.4)	597 (35.3)	
Number of hospitalizations for violence victimization experiences	≥5	303 (0.5)		303 (18.6)	<0.001
	3–4	484 (0.8)		484 (29.0)	
	1–2	889 (1.4)		889 (52.4)	
	None	60,600 (97.3)	60,600 (100)		

Weighted percentages following complex sample analysis.

**Table 2:** Prevalence of tobacco, alcohol, and drug use among Korean adolescents who have received hospitalization for violence victimization, stratified in terms of sex.

Variable	Boys (*n* = 31,624)			Girls (*n* = 30,652)		
	Non-Victim (*n* = 30,466)	Victim (*n* = 1158)	*p*	Non-Victim (*n* = 30,134)	Victim (*n* = 518)	*p*
**Tobacco use**						
Previous smoking	19.5	36.1	<0.001	6.4	33.6	<0.001
Current smoking	8.8	27.4	<0.001	2.7	24.9	<0.001
Daily smoking	4.4	11.0	<0.001	1.0	8.1	<0.001
Heavy smoking	0.5	9.3	<0.001	0.1	8.1	<0.001
Early initiation of smoking *	23.9	61.7	<0.001	20.8	54.3	<0.001
Previous EC use	11.0	28.2	<0.001	2.4	25.7	<0.001
Current EC use	2.7	19.3	<0.001	0.6	19.4	<0.001
Daily EC use	0.6	5.6	<0.001	0.1	6.0	<0.001
Early initiation of EC use **	7.2	57.3	<0.001	12.7	70.2	<0.001
**Alcohol use**						
Previous drinking	43.6	53.8	<0.001	35.7	55.4	<0.001
Current drinking	17.6	33.0	<0.001	13.4	32.8	<0.001
Daily drinking	0.3	6.4	<0.001	0.1	6.5	<0.001
Problematic drinking	12.0	19.0	<0.001	9.7	21.3	<0.001
Early initiation of drinking ***	26.3	57.7	<0.001	18.7	58.3	<0.001
**Drug use**						
Previous drug use	0.9	15.4	<0.001	0.5	17.9	<0.001
Current drug use	0.2	5.4	<0.001	0.1	7.5	<0.001
Diet pill use without a prescription	0.6	11.1	<0.001	1.7	9.3	<0.001

EC = electronic cigarette; * *n* = 8097 (6076 boys, 2021 girls); ** *n* = 4260 (3467 boys, 793 girls); *** *n* = 24,355 (13,583 boys, 10,772 girls).

**Table 3:** Prevalence of tobacco, alcohol, and drug use among Korean adolescents in terms of number of hospitalizations for violence victimization.

Number of Violence Victimization Experiences	None	1–2/year	3–4/year	≥5/year	*p*
	(*n* = 60,600)	(*n* = 889)	(*n* = 484)	(*n* = 303)	
**Tobacco use**					
Previous smoking	13.1	27.2	41.5	48.7	<0.001
Current smoking	5.8	18.2	32.6	41.4	<0.001
Daily smoking	2.8	6.4	8.7	22.8	<0.001
Heavy smoking	0.3	2.8	10.4	23.8	<0.001
Early initiation of smoking *	23.2	40.0	65.7	83.2	<0.001
Previous EC use	6.8	18.4	34.5	41.7	<0.001
Current EC use	1.7	10.2	26.8	33.5	<0.001
Daily EC use	0.3	1.7	5.3	17.6	<0.001
Early initiation of EC use **	8.1	40.0	71.4	73.5	<0.001
**Alcohol use**					
Previous drinking	39.8	48.7	58.3	63.9	<0.001
Current drinking	15.6	25.9	37.7	45.5	<0.001
Daily drinking	0.2	1.6	4.5	23.2	<0.001
Problematic drinking	9.1	21.7	38.1	43.8	<0.001
Early initiation of drinking ***	23.0	44.8	67.7	73.1	<0.001
**Drug use**					
Previous drug use	0.7	6.7	21.7	33.7	<0.001
Current drug use	0.1	1.7	6.7	17.2	<0.001
Diet pill use without a prescription	1.1	7.2	12.8	16.7	<0.001

EC = electronic cigarette; * *n* = 8097 (*n*_none_ = 7543, *n*_1–2/year_ = 233, *n*_3–4/year_ = 182, and *n*_≥5/year_ = 139); ** *n* = 4260 (*n*_none_ = 3842, *n*_1–2/year_ = 153, *n*_3–4/year_ = 152, and *n*_≥5/year_ = 113); *** *n* = 24,355 (*n*_none_ = 23,471, *n*_1–2/year_ = 427, *n*_3–4/year_ = 273, and *n*_≥5/year_ = 184).

**Table 4:** Association between substance use and history of hospitalization for violence victimization among Korean adolescents, stratified by sex.

Variable	Boys (*n* = 31,624)		Girls (*n* = 30,652)	
	Non-Victim (*n* = 30,466)	Victim (*n* = 1158)	Non-Victim (*n* = 30,134)	Victim (*n* = 518)
**Tobacco use**				
Previous smoking	1.00	2.07 (1.79–2.40)	1.00	4.77 (3.77–6.03)
Current smoking	1.00	3.44 (2.88–4.10)	1.00	6.18 (4.65–8.21)
Daily smoking	1.00	2.09 (1.67–2.63)	1.00	3.07 (1.98–4.76)
Heavy smoking	1.00	10.52 (8.02–13.79)	1.00	11.94 (6.56–21.74)
Early initiation of smoking *	1.00	4.37 (3.48–5.48)	1.00	3.12 (2.18–4.46)
Previous EC use	1.00	2.79 (2.39–3.24)	1.00	7.31 (5.50–9.72)
Current EC use	1.00	6.70 (5.52–8.14)	1.00	15.39 (10.56–22.43)
Daily EC use	1.00	5.64 (4.06–7.84)	1.00	10.99 (5.80–20.83)
Early initiation of EC use **	1.00	16.29 (11.95–22.20)	1.00	10.77 (6.25–18.58)
**Alcohol use**				
Previous drinking	1.00	1.37 (1.20–1.57)	1.00	1.82 (1.49–2.23)
Current drinking	1.00	2.07 (1.78–2.41)	1.00	2.51 (2.03–3.11)
Daily drinking	1.00	8.71 (6.21–12.21)	1.00	17.53 (8.06–38.12)
Problematic drinking	1.00	1.40 (1.16–1.70)	1.00	1.90 (1.48–2.43)
Early initiation of drinking ***	1.00	3.59 (2.94–4.39)	1.00	5.76 (4.12–8.05)
**Drug use**				
Previous drug use	1.00	11.55 (9.29–14.35)	1.00	13.78 (9.45–20.08)
Current drug use	1.00	11.88 (7.55–18.70)	1.00	14.45 (6.84–30.51)
Non-prescribed diet-pill use	1.00	14.26 (10.75–18.91)	1.00	3.57 (2.57–4.96)

EC = electronic cigarette; adjusted by grade, residential area, perceived school performance, economic status, perceived stress, depression, and suicidal ideation; * *n* = 8097 (6076 boys, 2021 girls); ** *n* = 4260 (3467 boys, 793 girls); *** *n* = 24,355 (13,583 boys, 10,772 girls).

**Table 5:** Association between substance use and number of hospitalizations for violence victimization among Korean adolescents.

Number of Hospitalizations for Violence Victimization	None	1–2/year	3–4/year	≥5/year	*p* for Trend
	(*n* = 60,600)	(*n* = 889)	(*n* = 484)	(*n* = 303)	
**Tobacco use**					
Previous smoking	1.00	1.88 (1.59–2.21)	3.20 (2.56–4.01)	4.68 (3.60–6.08)	<0.001
Current smoking	1.00	2.69 (2.21–3.27)	5.31 (4.14–6.82)	8.06 (6.13–10.60)	<0.001
Daily smoking	1.00	1.64 (1.22–2.20)	1.74 (1.22–2.48)	6.17 (4.37–8.71)	<0.001
Heavy smoking	1.00	4.51 (2.76–7.36)	14.60 (9.77–21.83)	32.51 (23.06–45.84)	<0.001
Early initiation of smoking *	1.00	1.88 (1.41–2.49)	6.02 (4.44–8.14)	12.73 (8.11–19.98)	<0.001
Previous EC use	1.00	2.28 (1.90–2.75)	4.82 (3.79–6.14)	7.11 (5.42–9.33)	<0.001
Current EC use	1.00	4.55 (3.52–5.88)	12.76 (9.86–16.52)	16.65 (12.18–22.78)	<0.001
Daily EC use	1.00	2.70 (1.50–4.87)	6.23 (3.91–9.90)	21.16 (13.94–32.14)	<0.001
Early initiation of EC use **	1.00	7.44 (5.13–10.80)	25.58 (16.28–40.18)	22.83 (13.19–39.53)	<0.001
**Alcohol use**					
Previous drinking	1.00	1.25 (1.09–1.44)	1.63 (1.32–2.02)	2.17 (1.66–2.83)	<0.001
Current drinking	1.00	1.67 (1.42–1.97)	2.51 (2.04–3.09)	3.57 (2.72–4.69)	<0.001
Daily drinking	1.00	3.65 (2.10–6.33)	7.54 (4.37–13.01)	36.44 (24.63–53.91)	<0.001
Problematic drinking	1.00	1.29 (1.07–1.56)	1.27 (0.96–1.67)	2.98 (2.19–4.05)	<0.001
Early initiation of drinking ***	1.00	2.10 (1.63–2.70)	7.51 (5.73–9.84)	8.91 (6.07–13.07)	<0.001
**Drug use**					
Previous drug use	1.00	6.07 (4.49–8.22)	20.10 (15.37–26.28)	25.40 (19.04–33.88)	<0.001
Current drug use	1.00	6.03 (3.11–11.71)	18.24 (11.07–30.05)	29.68 (18.33–48.04)	<0.001
Diet pill us without a prescription	1.00	6.10 (4.53–8.22)	10.46 (7.86–13.91)	11.04 (7.41–16.43)	<0.001

EC = electronic cigarette; Adjusted by sex, school grade, residential area, perceived school performance, economic status, perceived stress level, depression, and suicidal ideation; * *n* = 8097 (*n*_none_ = 7543, *n*_1–2/year_ = 233, *n*_3–4/year_ = 182, and *n*_≥5/year_ = 139); ** *n* = 4260 (*n*_none_ = 3842, *n*_1–2/year_ = 153, *n*_3–4/year_ = 152, and *n*_≥5/year_ = 113); *** *n* = 24,355 (*n*_none_ = 23,471, *n*_1–2/year_ = 427, *n*_3–4/year_ = 273, and *n*_≥5/year_ = 184).
